# Should the Arteriovenous Fistula Be Created before Starting Dialysis?: A Decision Analytic Approach

**DOI:** 10.1371/journal.pone.0028453

**Published:** 2011-12-07

**Authors:** Swapnil Hiremath, Greg Knoll, Milton C. Weinstein

**Affiliations:** 1 Division of Nephrology, Kidney Research Center, Ottawa Hospital Research Institute, Ottawa, Ontario, Canada; 2 Clinical Epidemiology Program, Ottawa Hospital Research Institute, Ottawa, Ontario, Canada; 3 Department of Health Policy and Management, Harvard School of Public Health, Boston, Massachusetts, United States of America; National Cancer Institute, United States of America

## Abstract

**Background:**

An arteriovenous fistula (AVF) is considered the vascular access of choice, but uncertainty exists about the optimal time for its creation in pre-dialysis patients. The aim of this study was to determine the optimal vascular access referral strategy for stage 4 (glomerular filtration rate <30 ml/min/1.73 m^2^) chronic kidney disease patients using a decision analytic framework.

**Methods:**

A Markov model was created to compare two strategies: refer all stage 4 chronic kidney disease patients for an AVF versus wait until the patient starts dialysis. Data from published observational studies were used to estimate the probabilities used in the model. A Markov cohort analysis was used to determine the optimal strategy with life expectancy and quality adjusted life expectancy as the outcomes. Sensitivity analyses, including a probabilistic sensitivity analysis, were performed using Monte Carlo simulation.

**Results:**

The wait strategy results in a higher life expectancy (66.6 versus 65.9 months) and quality adjusted life expectancy (38.9 versus 38.5 quality adjusted life months) than immediate AVF creation. It was robust across all the parameters except at higher rates of progression and lower rates of ischemic steal syndrome.

**Conclusions:**

Early creation of an AVF, as recommended by most guidelines, may not be the preferred strategy in all pre-dialysis patients. Further research on cost implications and patient preferences for treatment options needs to be done before recommending early AVF creation.

## Introduction

The burden of chronic kidney disease (CKD) continues to increase, with 571,414 patients in the end-stage renal disease (ESRD) program in the United States in 2009 [1]. The majority of these patients, 398,861, are on hemodialysis. An even greater number of patients have advanced kidney failure with a glomerular filtration rate less than 30 ml/min/1.73 m^2^ (Stage 4 CKD) [2]. In the United States alone, it is estimated that 0.35% of the adult population has stage 4 CKD, which translates into more than 800,000 people. In 2009, 116,395 CKD patients progressed to ESRD and started hemodialysis in the United States [1].

The arteriovenous fistula (AVF) has been identified as the optimal vascular access for hemodialysis patients based on improved survival and fewer complications as compared to arteriovenous grafts (AVG) and tunneled central venous catheters (CVC) [3]. Despite this, more than 80% of incident hemodialysis patients start with a CVC as their vascular access [1]. Timely creation of an AVF before the need for dialysis therapy may allow adequate time for the fistula to mature as well as provide sufficient time to perform another vascular access procedure if the first attempt fails, thus obviating the need for a CVC, though firm evidence for the same is lacking [4], [5]. Hence, most guidelines recommend assessment of patients for access creation at the CKD 4 stage [5]–[9].

However, early AVF creation is not without problems. A small number of patients may develop ischemic steal syndrome from arterial ischemia in the distal limb or develop high output heart failure. Both of these complications usually require AVF ligation [10], [11]. In addition, early AVF creation, prior to dialysis, will likely result in many patients undergoing unnecessary surgery since most stage 4 CKD patients are much more likely to die than to actually develop ESRD and require dialysis [12]. Lastly, greater than 25% of AVF may never mature enough to be used functionally [13].

Thus creation of an AVF when a patient has stage 4 CKD but is not yet on dialysis has both risks and potential benefits. There are no validated prediction models to determine which patients will progress to ESRD and thus should have an AVF created. Therefore, patients in stage 4 CKD have two options; they can either proceed with early AVF creation or start dialysis with a CVC and proceed with AVF later. We used a decision- analytic model to compare these two treatment options faced by patients with stage 4 CKD. The model estimated survival as well as quality-adjusted survival.

## Methods

### The Decision Model

We used a Markov model to compare two treatment strategies for stage 4 CKD patients: (1) AVF strategy and (2) Wait strategy. In the model, hypothetical cohorts of patients are followed for the remainder of their lifetimes [14]. With each monthly ‘cycle’ of the model, patients may move between several different health states (e.g. CKD stage 4 with no AVF, CKD stage 4 with AVF, Dialysis with CVC, Dialysis with AVF, death) according to the occurrence of clinical events (e.g. progression to dialysis, development of heart failure due to AVF, etc). The probabilities that each of these events occurs was determined using the best available data from the literature. Because some of the transition probabilities depend on the time since entering a state (such as mortality after starting dialysis), we created “tunnel” states which are essentially copies of a state that track the length of time spent in the state [15].

By simulating outcomes in large numbers of identical patients, the average accumulated survival time with the two treatment strategies may be estimated. For our base case analysis, we chose a 70-year-old patient with CKD stage 4, because the 65–74 year age group is the fastest growing segment in the dialysis population [1]. It also represents a cohort where clinical equipoise regarding the optimal strategy is the greatest [16].

**Figure 1:**
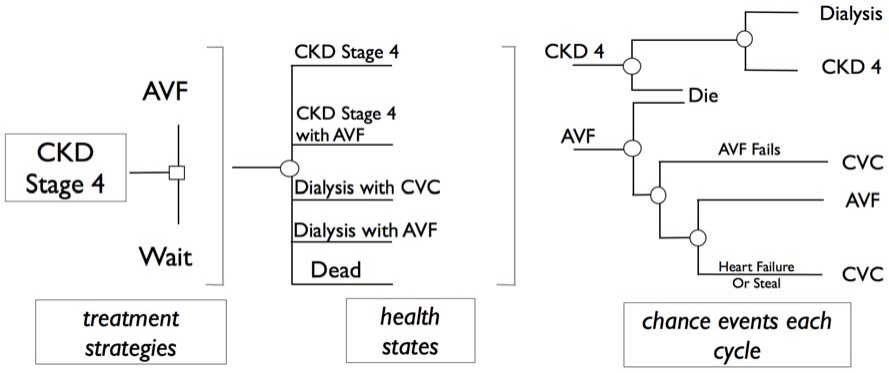
Schematic representation of the decision-analysis model.

Our decision model (Figure 1) evaluated the following two treatment strategies:

1. AVF Strategy: CKD stage 4 patients get referred for an AVF; and
2. Wait Strategy: CKD stage 4 patients are not referred for an AVF. When they reach the point of starting dialysis, they get a CVC as their vascular access and are then referred for AVF surgery.

During each cycle of the model (1 month in this analysis), hypothetical patients in any given health state are at risk of several events, which may result in transitions to other health states. For certain health states, we created “tunnel” states to force patients to remain in that state for a fixed number of cycles (e.g. to account for a three-month maturation period for AVF). Stage 4 CKD patients may progress to dialysis; CKD or dialysis patients with an AVF may develop heart failure or ischemic steal syndrome; patients with a CVC may develop central vein stenosis; death may occur while they are still in CKD stage 4 or while they are on dialysis.

### Assumptions

The base case was that of a 70 year old man with CKD stage 4. We assumed that the only choice of renal replacement therapy (RRT) for this patient was hemodialysis; peritoneal dialysis and renal transplantation were not considered in this analysis.

*In the AVF strategy:*
  1. All patients with CKD stage 4 would be referred for an AVF; it would take 2 months to create an AVF and 3 months for it to mature and be functional.
  2. If an AVF failed, another attempt to make an AVF would not be made.
  3. If a patient with an AVF developed CHF or steal, the AVF would be ligated in the same month. Hence heart failure and steal syndrome were modeled as temporary health states.
*In the wait strategy:*
  1. All CKD 4 patients progressing to need dialysis would start with a CVC.
  2. Once on dialysis, they would all be referred to get an AVF, with the same waiting and maturation period as above.
  3. During the 2 cycles of waiting to get AVF surgery, a proportion of patients would decide not to have an AVF and remain on dialysis with a CVC.

### Probabilities

Estimates and plausible ranges of event probabilities were obtained from published articles and expert opinion (Table 1). Both deterministic threshold analyses and probabilistic sensitivity analyses were performed, as described below.

**Table 1:** Probabilities and Utilities.

Variables	Best Estimate	Range (for sensitivity analysis)	Distribution	Reference
Rate of progression to ESRD	0.0076	0.00356–0.019	Lognormal	19
CKD stage 4 mortality	0.0097	0.00375–0.0279	Lognormal	19
Mortality on dialysis with CVC in first three months	0.042	0.0126–0.0503	Lognormal	27
Mortality on dialysis with CVC after three months	0.020	0.0126–0.0503	Lognormal	27
Mortality on dialysis with AVF in first three months	0.018	0.0061–0.0232	Lognormal	27
Mortality on dialysis with AVF after three months	0.013	0.0061–0.0232	Lognormal	27
Patient refusal for an AVF	0.0467	0.01–0.1	Beta	29
Central vein stenosis	0.017	0.001–0.05	Beta	29
Heart Failure due to AVF	0.0004	0.001–0.09	Lognormal	Expert opinion
Mortality due to heart failure	0.012	0.01–0.5	Beta	Expert Opinion
Surgical mortality	0.001	0.0001–0.005	Beta	Expert opinion
Ischemic steal syndrome	0.0504	0.001–0.09	Lognormal	29
AVF Failure: first three months	0.025	0–0.9	Lognormal	28
AVF Failure: after three months	0.016	0–0.9	Lognormal	28
AVF failure on dialysis	0.010	0–0.9	Lognormal	28
Utility of CKD stage 4 without AVF	0.62	0.40–0.84	Triangular	31
Utility of CKD stage 4 with AVF	0.62	0.40–0.84	Triangular	31
Utility of dialysis	0.51	0.20–0.82	Triangular	31

We identified 8 studies in the literature on mortality and progression to ESRD for stage 4 CKD patients [12], [17]–[23] (Table 2). The rates of progression varied from a low of 4.27 per 100 person-years from a cardiac database [12] that likely had many patients with ischemic nephropathy, to a high of 14.3 per 100 person-years [20] from a nephrology database that had a significant proportion of proteinuric patients at high risk for progression. For our base case we used data from O'Hare et al [21] (9.31 per 100 person-years), since they provided data for patients aged 65 to 74 years. The lowest and highest rates of progression from the 8 studies were used in the sensitivity analyses. The mortality rate for stage 4 CKD patients ranged from a low of 4.5 per 100 person-years to a high of 33.45 per 100 person years in the same 2 studies [12], [20]. As for progression, we used data from the O'Hare study [21], (mortality rate 11.68 per 100 person-years) with the extreme values used for sensitivity analyses.

**Table 2:** Summary of literature on mortality and progression to ESRD in CKD stage 4.

Study	Mean age (years)	GFR (ml/min)	Progression to ESRD	Mortality	Population
Keith (2001)	73.6±13.6	15–29	19.9%*	45.7%*	Large HMO
Go (2004)	70.1±14.5	15–29		11.36 per 100 PY‡	Large HMO
Patel (2005)	70.0±10.0	15–29	14.2 per 100 PY	20.1 per 100 PY	Veterans
O'Hare (2007)	65–74	15–29	9.31 per 100 PY	11.68 per 100 PY	Veterans
O'Hare (2007)	75–84	15–29	6.31 per 100 PY	15.39 per 100 PY	Veterans
Roderick (2009)	83.2±7.1	<30		19–29 per 100 PY	UK General Practice
Keough-Ryan (2008)	69.2±13.2	<30	4.27 per 100 PY	33.45 per 100 PY	Post acute cardiac event
Levin (2008)	66.8±14.5	<30	14.3 per 100 PY	4.5 per 100 PY	Referred population
Conway (2009)	Median 71.6	<30	3.8%†	10.4%†	Referred population

*crude data in percentages, over 66 months of follow up.

† crude 1 year data in percentages.

‡ Age-Standardized Rates.

PY: patient-years.

HMO: Health Maintenance Oraganization.

Six studies were identified which reported on mortality rates for patients starting dialysis with a CVC or fistula [24]–[29] (Table 3). The two studies in prevalent patients were published in 2001 and 2002, comprised younger patients (mean age <60 yrs), and had mortality rates of 7.29 to 13 per 100 person-years for patients with an AVF and 15.16 to 23 per 100 person-years for patients with CVC [25], [27]. In contrast, the more recent studies included older, incident patients with higher mortality rates [24], [26], [28], [29]. The mortality rate in patients with CVC, however, was approximately 1.5 to 2 times that of patients with an AVF in all the studies [24]–[29]. We used data from Xue et. al. for our base case, since that study provided separate mortality rates according to access type for the first 90 days of dialysis and thereafter as well as included elderly patients in the 65–74 age range [29]. A tunnel state was created to account for the fact that the mortality rate is significantly higher in the first three months of dialysis before it levels off.

**Table 3:** Summary of literature on difference in mortality with CVC and AVF.

Study	Mean age	Mortality with AVF (per 100 PY)	Mortality with CVC (per 100 PY)	Population
Dhingra (2001) Diabetes	59.2	13*	22*	Prevalent, DMMS Wave 1
Dhingra (2001) No Diabetes	59.2	11*	23*	Prevalent, DMMS Wave 1
Pastan (2002)	58.3±0.2	7.29	15.16	Prevalent ESRD Network 6
Xue (2003) First 90 days	∼75	28.8	60.4	Incident Medicare
Xue (2003) Next 9 months	∼75	21.6	52.8	Incident Medicare
Polkinghorne (2004)	61 (range 48–71)	8.6	26.1	Incident
Moist (2008)	68 (median)		HR 1.6†	Incident
Bradbury (2009)	62.5±15	9.96‡	53.62‡	Incident, DOPPS I & II

*Adapted from Adjusted patient survival data.

† Hazard ratio, compared to mortality with AVF.

‡ Six month follow up data.

PY: patient-years.

Rates of fistula failure were obtained from a Dutch prospective study [30]. Fistula failure rates due to the loss of fistula patency before cannulation were used to model failure rates in the CKD stage. Functional failure, which refers to loss of fistula patency after cannulation, was used to model failure rates in the dialysis states. We incorporated secondary fistula failure rates, which include intervening manipulations designed to re-establish or maintain the functionality. Tunnel states were created to account for the high fistula failure rates in the first 3 months after creation which plateau thereafter. The probabilities of developing steal syndrome and central vein stenosis, and of refusing dialysis, were derived from a cross-sectional study [31]. Since there were only case reports and no summary estimates of the incidence rate of high output heart failure with AVF, it was assumed to be 5 per 1000 patient-months based on expert opinion (SH, GK). Since there were no data on operative mortality rate for an AVF creation or ligation surgery, it was similarly assumed to be 1 in 1000 based on expert opinion (SH, GK). Both of these subjective estimates were subjected to sensitivity analysis.

### Outcomes

Life expectancy with each strategy was calculated based on the average accumulated survival time. Quality-adjusted life expectancy was calculated by weighting the time spent in each state with the preference-based utility of that state [32]. The utilities for the health states of CKD stage 4 and dialysis were obtained from a Canadian study that measured the Short Form-6D (SF-6D) and Health Utilities Index Mark 3 (HUI), the latter of which was used [33]. We assumed that an AVF would not result in a significant disutility for CKD stage 4 patients. We also assumed that utilities for dialysis patients with CVC and AVF would be the same; sensitivity analyses were performed to test these assumption. Utilities assigned to each month were the average of those for the patient's health state at the beginning and end of the month [34].

### Analysis

The analysis was done using a Markov cohort method with 100,000 patients. Model verification (debugging) was done by building up the model from simple to more complex, checking each step visually, examining the state probabilities from the cohort analysis, exploring certain extreme values and doing a sensitivity analysis on all variables. The model was calibrated by comparing simulated events (mortality for dialysis patients in the model) to observed ones (from the USRDS report) [1]. A probabilistic sensitivity analysis was done by assigning probability distributions around model parameters and by using Monte Carlo simulation [35] (table 4). All analysis was done using TreeAge Pro 2008 software (version 1.3.1, Williamstown, MA) and JMP (version 8.0, SAS Inc., Cary, NC).

**Table 4:** Probability Distributions and parameter estimates used in the Probabilistic Sensitivity Analysis.

Variables	Distribution	Parameters
Rate of progression to ESRD	Lognormal	μ = −2.333;σ = 0.406
CKD stage 4 mortality	Lognormal	μ = −2.577;σ = 0.415
Mortality on dialysis with CVC in first three months	Lognormal	μ = −5.473;σ = 0.604
Mortality on dialysis with CVC after three months	Lognormal	μ = −6.215;σ = 0.759
Mortality on dialysis with AVF in first three months	Lognormal	μ = −6.320;σ = 0.901
Mortality on dialysis with AVF after three months	Lognormal	μ = −6.645;σ = 0.724
Patient refusal for an AVF	Beta	r = 28;n = 599
Central vein stenosis	Beta	r = 10;n = 599
Heart Failure due to AVF	Lognormal	μ = −9.210;σ: 0.601
Mortality due to heart failure	Beta	r = 5;n = 404
Surgical mortality	Beta	r = 1;n = 1000
Ischemic steal syndrome	Lognormal	μ = −2.987;σ = 0.768
AVF Failure: first three months	Lognormal	μ = −3.689;σ = 1.010
AVF Failure: after three months	Lognormal	μ = −4.135;σ = 0.970
AVF failure on dialysis	Lognormal	μ = −4.605;σ = 1.177
Utility of CKD stage 4	Triangular	low = 0.40; most likely = 0.62; high = 0.84
Utility of dialysis	Triangular	low = 0.20; most Likely = 0.51; high = 0.82

## Results

### Base Case Analysis

The results of the base case analysis showed that the wait strategy resulted in a slightly higher life expectancy (66.55 vs 65.9 months) and quality-adjusted life expectancy (QALE) (38.89 vs. 38.49 quality-adjusted life months) as compared to the AVF strategy (Table 5).

**Table 5:** Results of base case analysis.

Strategy	Life expectancy (in months)	Gain in life expectancy	Quality adjusted life expectancy (in months)	Gain in quality adjusted life expectancy
Wait	66.55	0.65	38.89	0.50
AVF	65.90	-	38.49	-

### Sensitivity Analysis

**Figure 2:**
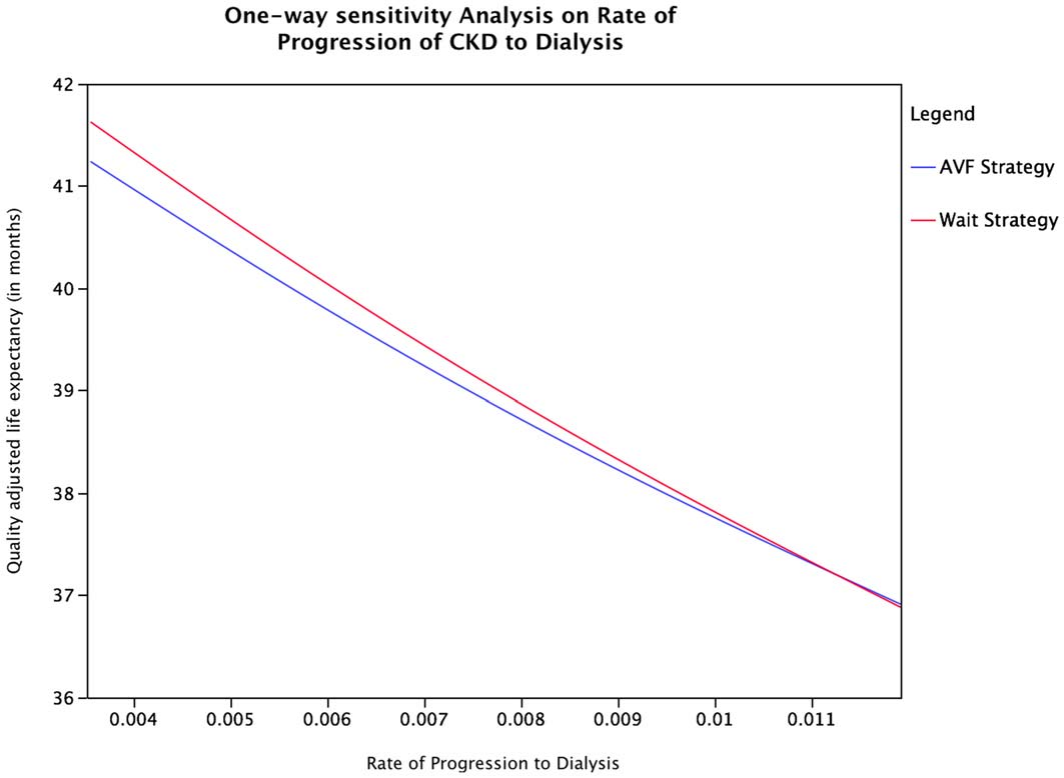
One way sensitivity analysis based on rate of progression of CKD stage 4 to dialysis: This demonstrates that the wait strategy results in a higher quality-adjusted life expectancy at lower rates of progression and the AVF strategy results in a higher quality adjusted life expectancy at higher rates of progression of CKD to dialysis.

Multiple one-way sensitivity analyses were carried out for all the variables entered in the model. The optimal strategy changed at very high rates of progression of CKD to dialysis as well at lower rates of steal syndrome than used in the base case analysis. When the rate of progression was higher than 0.01126 (corresponding to 14.5 per 100 patient-years), the optimal strategy was to refer patients for AVF creation (Figure 2). The additional LE and QALE obtained at the highest rate of progression used in the sensitivity analysis were 0.05 and 0.03 months respectively. Similarly, the optimal strategy changed to AVF creation when the probability of steal syndrome was lower than 0.023. The additional LE and QALE obtained with the AVF strategy when there was no steal syndrome were 0.5 and 0.3 months respectively. If the utility of CKD stage 4 patients with an AVF was higher than 0.7 whilst maintaining the utility of CKD stage 4 patients without an AVF at 0.62, the optimal strategy reverted to the AVF strategy. When the fistula failure rates were changed to zero, the optimal strategy did not change. The optimal strategy is also otherwise robust across the range of probabilities tested for all other parameters (Table 1).

**Figure 3:**
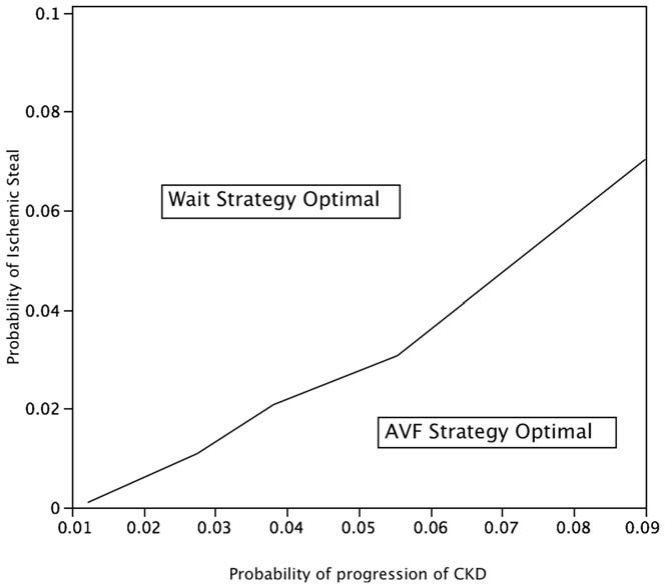
Two-way sensitivity analysis plotting rate of progression to dialysis and probability of steal: This demonstrates that the wait strategy results in a higher quality-adjusted life expectancy at lower rates of progression and lower probablility of ischemic steal and the AVF strategy results in a higher quality adjusted life expectancy at higher rates of progression of CKD to dialysis and higher rates of ischemic steal.

A two-way sensitivity analysis with respect to the probabilities of progression and steal syndrome is shown in Figure 3. It demonstrates that as the rate of progression to dialysis increases, the AVF strategy becomes optimal despite increasing probability of steal syndrome.

**Figure 4:**
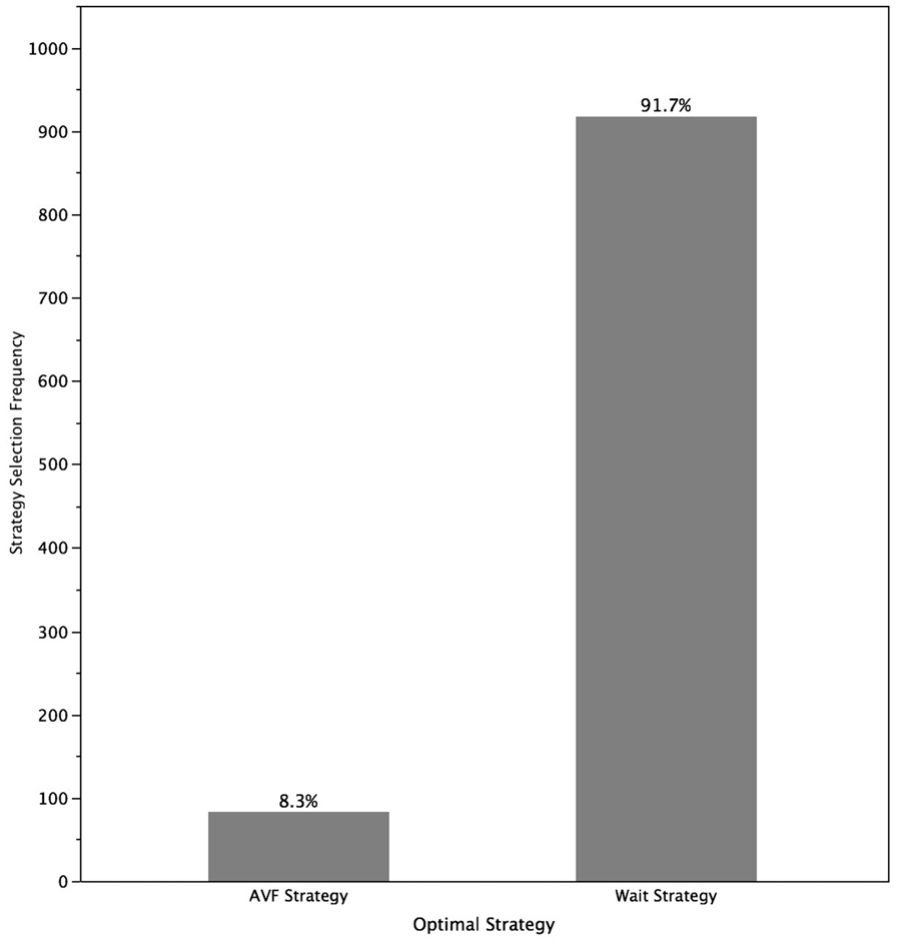
Incremental outcomes and strategy selection frequency with the probabilistic sensitivity analysis using a Monte Carlo simulation.

Incremental outcomes from the probabilistic sensitivity analysis, expressed as a difference of quality adjusted life expectancy obtained between the two strategies, were obtained using a Monte Carlo simulation. The probability that the wait strategy is optimal was 91.7% (Figure 4).

## Discussion

In this decision analysis, we have shown that the wait strategy is the optimal strategy for our base case of a 70-year old patient with stage 4 CKD. However, the gains in life expectancy and quality-adjusted life expectancy are likely to be less than one month. The analysis was robust across the range of values for most variables in the model, with the possible exception of the rate of progression to ESRD and the rate of steal syndrome.

These results suggest that recommendations of the Fistula First Breakthrough Initiative and the various society guidelines, to consider early creation of AVF in the predialysis period, may not apply all patients. It might especially be prudent to wait in patients similar to our base case, who have a slow rate of progression and high rates of competing events. Indeed, it may not be optimal to wait in patients with a high rate of progression (such as proteinuric diabetic nephropathy) though the quality-adjusted life expectancy gained by early fistula creation in such patients is less than one month. Additionally, the assumption underlying these conclusions is that the patients who wait will get an AVF soon after initiation of dialysis, and will not be exposed to the deleterious effects of a CVC beyond 3 months. The strategy of initiating dialysis with a catheter in appropriate patients has been suggested before [36] and our study helps to quantify the benefit of such a decision. Also, conversion from a CVC to AVF has been reported to be associated with an improved survival [24] and hospitalization risk is also higher for patients who continue with a CVC compared to those who convert from a CVC to an AV access [37]. It could be argued that patients who start dialysis with a CVC may not want to have another surgery for AVF creation, but we incorporated that by inserting a parameter for higher patient refusal for AVF in the wait strategy.

In the present study, we are not suggesting that patients should start with and remain on a CVC, or that CVCs are superior to AVFs as vascular access. It has been suggested, however, that in the elderly population, and in octogenarians specifically, that an assessment of life expectancy and quality of life should be made while planning for vascular access [36], [38].

The model construction itself had some limitations. We considered only hemodialysis as an option for our base case analysis. While this is the most common initial treatment modality for patients fitting our base case [39], some patients will indeed opt for peritoneal dialysis or kidney transplantation. This may limit the generalizability of our findings to all stage 4 CKD patients. We also assumed that only one attempt would be made to create an AVF. This assumption resulted in most patients having a failed AVF by the time they started hemodialysis. In clinical practice, it could be argued that many of these patients would have had a second attempt at AVF creation. In our cross-sectional study, we found that a median of two attempts (range, 1 to 4) were made at AVF creation [31]. However, in sensitivity analysis, the wait strategy remained optimal despite an AVF failure rate of zero, suggesting that incorporating multiple attempts will not change the result. Lastly, we did not include AV grafts (AVG) as an option for vascular access. AVGs have been compared to AVFs in a decision analysis and were reported to have a lower median survival, albeit less than expected, by 2.6 months [40].

There were limitations related to the data sources used in the model. Since no randomized controlled trial has ever been done to compare outcomes between AVF and CVC in dialysis patients, we had to rely on data from administrative sources to obtain estimates of mortality rates in patients with CVC and AVF. However, many incident dialysis patients with a CVC in these databases may have been patients with acute renal failure in whom the mortality rate may be much higher than for patients with known renal disease and a planned start with a CVC. More accurate data reflecting lower mortality with CVC would, however, make the results favoring the wait strategy even stronger. We used post-intervention figures from a Dutch study in our estimates of fistula failure rates [30]. These figures might be optimistic in North American situations since European centers have a significantly higher prevalence of AVF compared to CVCs [41]; however lower fistula survival rates would make the wait strategy even more favorable. We chose wait times for access creation based on local data, however this may vary at other centres and novel approaches such as direct nephrologist selection for operation have resulted in shorter waiting times for access creation [42].

There were limitations related to the utilities in the model. We assumed that the utility for a patient with CKD stage 4 with an AVF would be the same as that of a patient with CKD stage 4 who did not have a fistula. Although the optimal strategy would change if having an AVF increased the utility for a CKD stage 4 patient, this is an unlikely scenario. In addition, we assumed that the utility of a dialysis patient with a CVC would be the same as for a dialysis patient with an AVF. Indeed, despite nephrologists' opinion of the AVF being the optimal access, interviews with patients who have refused an AVF suggest that they do not always focus on long-term mortality benefit, but rather day-to-day use and quality of life with a vascular access [43]. Although sensitivity analyses conducted on this parameter was robust, further studies are needed to correctly elucidate preference utilities in CKD and dialysis patients with different vascular access.

We did not assess the comparative costs of the two strategies, nor did we perform an incremental cost-effectiveness analysis. Because of the higher number of surgeries in the AVF strategy and the fact that the cost of an AVF surgery is higher than that of CVC insertion, it is likely that the cost would be higher in the AVF strategy, thus making the wait strategy dominant (more QALYs at lower cost) over the AVF strategy.

In summary, this analysis suggests that the optimal strategy in a typical elderly stage 4 CKD patients should be to wait and start with a CVC when required followed by AVF creation. This strategy was robust across most sensitivity analysis. However, it might not be optimal for patients with a very high probability of progression to dialysis, such as patients with proteinuric diabetic nephropathy. Further studies should be done to obtain more precise estimates of progression and develop prediction rules for progression of renal failure in CKD stage 4 which take competing events of death into account.
